# The Impact of Treatment with Beta-Blockers upon Mortality in Chronic Heart Failure Patients

**DOI:** 10.3889/oamjms.2016.022

**Published:** 2016-02-08

**Authors:** Borjanka Taneva, Daniela Caparoska

**Affiliations:** 1 *University Clinic of Cardiology, Ss Cyril and Methodius University of Skopje, Skopje, Republic of Macedonia*; 2 *University Clinic of Toxicology and Urgency Medicine, Ss Cyril and Methodius University of Skopje, Skopje, Republic of Macedonia*

**Keywords:** heart failure, beta-blockers, mortality, combined outcome, relative risk reduction

## Abstract

**BACKGROUND:**

Besides the conventional therapy for heart failure, the diuretics, cardiac glycosides and ACE-inhibitors, current pharmacotherapy includes beta-blockers, mainly because of their pathophysiological mechanisms upon heart remodeling.

**AIM:**

The study objective was to assess the cardiovascular mortality in the beta-blocker therapy group and to correlate it with the mortality in the control group as well as to correlate the combined outcome of death and/or hospitalization for cardiovascular reason between the two groups.

**MATERIALS AND METHODS:**

The study included 113 chronic heart failure patients followed up for a period of 18 months. The therapy group received conventional therapy plus the target dose of beta blockers, and the control group received the conventional therapy only. The therapy group was divided in three separate subgroups in terms of the type of beta-blocker (Metoprolol subgroup, Bisoprolol and Carvedilol subgroup). To compare the mortality and the combined outcome, the RRR (relative risk reduction) and NNT (number needed to treat) were used, as well as the survival analysis by Kaplan-Meier.

**RESULTS:**

The results showed the following: in regards of the cardiovascular mortality, the relative risk for death in the therapy group was 34%, which, though statistically not significant, is of great clinical significance. In regards of the combined outcome (death and/or number of hospitalizations) the results showed a RRR of 40% in the therapy group compared to the control group, which is statistically highly significant.

**CONCLUSION:**

The study confirmed that patients with stable chronic heart failure, treated with optimal doses of beta-blockers, show a significant reduction of the risk from death as well as combined outcome (death and/or number of hospitalizations).

## Introduction

Heart failure is a pathophysiological condition when the abnormal heart function results in a failure of the heart to achieve blood output adequate to meet the requirements of the tissue metabolism.

In condition of heart failure as a response to the heart dysfunction, several neuro-endocrine systems are activated, as well as the sympathetic nervous system. Norepinephrine and plasma-catecholamine levels are increased and correlate with mortality rate, but the density of myocyte beta-1 receptors is lowered in heart failure patients. Beta-blockers inhibit the activity of norepinephrine and enhance the density of beta-1 receptors. By lowering the heart rate they lower the oxygen demand, prolong the diastole, resulting in a better myocardial perfusion and less malignant arrhythmias. They protect the heart of the direct cardiotoxicity of the catecholamines [1, 2]. They also suppress several activated neurohumoral systems in heart failure: rennin-angiotensine system and endothelyne-1 system, a powerful vasoconstrictor [3].

Metoprolol and Bisoprolol are beta-1 selective, and Carvedilol is a potent antagonist of beta-1, beta-2 and also alfa-1 receptors. It defers from the other beta-blockers with the effect upon the polymorphonuclear cells and its antioxidant activity [4].

Beta–blockers are recommended for treatment of all patients with stabile heart failure from ischemic or non-ischemic etiology and reduced left ventricular ejection fraction in NYHA Class II to IV, as a standard therapy, including ACE-inhibitors and diuretics.

Despite their long term benefit they can make an initial worsening of the symptoms, and therefore, we should start their application carefully, starting with a low dose and gradually raising it to the target level [5].

The aims of the study were: to assess mortality rate in the group treated with the target dose of beta-blockers (the therapy group), compared with the group on conventional therapy (the control group). To compare the combined outcome (mortality and/or number of hospitalizations from cardiovascular reasons) between the two groups; to compare mortality as well as the combined outcome, regarding different types of beta-blockers in the therapy group (Metoprolol, Bisoprolol and Carvedilol); and to compare the mortality of each of the subgroups from the therapy group with the mortality in the control group.

## Material and Methods

One hundred and thirty five patients with verified heart failure were investigated in the Outpatient department of the Cardiology Clinic in a period of two years. Out of 135, 113 patients underwent a complete investigation, and 22 of them were excluded from the study due to a worsening of the condition in the titration period.

The follow up period of these 113 patients was 18 months. The minimum follow up period was 3 months. The patients were divided in 2 groups, statistically not different in age and gender. The first group was treated by conventional therapy and target dose of beta-blocker (the therapy group), and the second by conventional therapy only (the control group).

The therapy group was divided in 3 subgroups according to the type of beta-blocker: a subgroup with Metoprolol, Bisoprolol and Carvedilol.

Inclusion criteria: Age 40-70 years; Verified stable chronic heart failure: clinically according to the classification of the NYHA Functional Class from II –IV, and by echocardiography with a confirmed ejection fraction (EF%) of 45% or less.

The patient was required to be stable more than one month before included in the study.

The investigation included: complete laboratory analyses every 3 months, electrocardiogram once a month, one and two-dimensional transthoracic echocardiography every 3 months.

We compared mortality and the combined outcome between the two groups. To simplify the combined outcome we quantified this parameter by scoring other parameters taking part in it: (1) Number of hospitalizations (each hospitalization is scored by 1); and (2) number of acute attacks of chronic heart failure (each attack is scored by 1).

The study was clinical, prospective, interventional and controlled. We investigated 38 variables and compared the data of the 2 groups at the beginning of the follow up (the zero time) and at the end of follow up, using Student t-test for numerical and Chi square test for nominal parameters. To compare the variables of interest between the three subgroups in the therapy group, we used ANOVA-one way test. We calculated the relative risk reduction and the number needed to treat to prevent the outcome in one patient (RRR and NNT) for the variables of the primary objective (mortality and the combined outcome-mortality and number of hospitalizations from cardiovascular causes). We made an analysis of the complete survival of all the groups and compared the survival between the control and therapy group and all the therapy subgroups separately with the Kaplan-Meier method. A p value of < 0.05 was taken to be statistically significant.

## Results

Out of a total of 113 patients with chronic heart failure with NYHA-FC II–IV in a stable clinical condition, 60 were with a non-ischemic and 53 with ischemic heart failure. Ninety one of them were men and only 21 women (81.5% and 19.55% respectively), with a mean age of 57.3 ± 8.6 years. During the follow up the total mortality was 15 (13.2%).

The total patient population is divided in 2 groups, control and therapy group. The therapy group is divided in 3 subgroups, Metoprolol, Bisoprolol and Carvedilol.

When comparing the parameters in the total population group between the survived and the patients who died, at zero time and at the end of the follow up, there was a statistically lower ejection fraction at the end of the follow up in the group of the patients who died, as well as lower ΔEF%, higher number of hospitalizations, more frequent attacks of acute heart failure, higher NYHA-FC at the beginning and at the end of the follow up and also higher NYHA-score at the end of follow up, but regarding systolic and diastolic blood pressure, the values were statistically lower in the group of patients who died.

*CER* = the number of patients who died divided by the number of the patients in the control group.

*EER* = the number of patients who died divided by the number of the patients in the therapy group.

*95%CI (confidence interval)* is the interval (the borders) between the events.

CER= n (events)/total n EER= n (events)/total n

RR= EER/CER RRR %= (CER-EER)/CER x 100

NNT= 1/(CER-EER)

Regarding mortality rate of the patients with the target dose of beta-blockers, compared to the control group, there wasn’t any statistically significant difference (Chi square test), but the RRR%, although the value of 34% was also statistically not significant, it had a substantial clinical value.

Regarding the combined outcome (mortality and/or hospitalization), the therapy group showed a statistically significant improvement (p < 0.0008). RRR% was 40% and NNT was 3.03, which indicates that if we treat 3 patients with a target dose of beta-blockers, we might prevent the combined outcome in one patient.

The mortality in the 3 therapy subgroups did not show a statistically significant difference when compared to the control group, but the combined outcome (mortality and/or hospitalizations) in the 3 subgroups, separately, when compared to the control group, showed a statistically significant improvement.

Regarding RRR% and NNT, there was a major improvement for both outcomes in all the 3 subgroups of beta blockers, but the Carvedilol subgroup showed highest values for RRR% (mortality - 56%, and the combined outcome - 38%) (Table 4, 5, 6).

**Table 1:** Clinical and laboratory parameters for the total patient population (control and therapy group)

Variable	n/M ± SD
Gender: men women	91 (80.5%) 22 (19.5%)
Age	57.35 ± 8.6
Weight (in zero time) - kg	76.18 ± 11.6
Weight (end of follow up) kg	70.81 ± 12.3
BMI (in zero time) kg/m^2^	26.11 ± 2.8
BMI (end of follow up)	24.4 ± 12.5
Htc (in zero time) vol%	0.39 ± 0.05
Htc (end of follow up)	0.37 ± 0.05
Scr (μmol/l)	84.53 ± 8.4
Alb (g/l)	44.01 ± 3.1
Total. lipids (g/l)	8.87 ± 1.3
HDL (mmol/l)	1.13 ± 0.2
LDL (mmol/l)	3.51 ± 0.7
Triglicerids (mmol/l)	1.53 ± 0.5
Na (mmol/l)	141.7 ± 2.6
K (mmol/l)	4.65 ± 0.4
ECG-zero time	1.66 ± 1.15
ECG-end of follow up	1.82 ± 1.17
EF % (zero time)	36.79 ± 6.6
EF % (end of follow up)	37.3 ± 8.3
ΔEF%	1.17 ± 6.8
NYHA-FC(zero time)	3.27 ± 0.7
NYHA-FC(end of follow up)	2.55 ± 0.9
NYHA score	0.35 ± 0.55
Number of hospitalizations	1.0 ± 1.26
Number of attacks of AHF	0.57 ± 0.98
SBP (mmHg)	98.45 ± 15.9
DBP (mmHg)	65.25 ± 9.7
Diagnosis:Ischemic nNon-ischemic HF	53 (46.9%) 60 (53.1%)
Mortality	15 (13.2%)

**Table 2:** Comparison of the parameters between the group of patients who died and the survived ones

Parameters	Survived n=98 M ± SD	Dead n=15 M ± SD	P =
Weight - zero time (kg)	77.86 ± 12.55	76.46 ± 9.93	0.68
Weight - end of follow up	72.54 ± 11.74	69.33 ± 11.65	0.32
Htc - zero time (vol%)	0.396 ± 0.5	0.402 ± 0.4	0.64
Htc end of follow up	0.377 ± 0.5	0.366 ± 0.4	0.49
Scr (μmol/l)	84.47 ± 8.41	88.96 ± 6.11	0.49
Alb (g/l)	44.28 ± 2.67	43.56 ± 4.73	0.39
Tlip (g/l)	8.82 ± 1.22	8.84 ± 1.24	0.96
HDL(mmol/l)	1.22 ± 0.89	1.14 ± 0.22	0.74
LDL (mmol/l)	3.47 ± 0.63	3.47 ± 0.66	0.98
Tg (mmol/l)	1.55 ± 0.47	1.60 ± 0.55	0.70
Na (mmol/l)	141.82 ± 2.45	140.65 ± 3.52	0.10
K (mmol/l)	4.55 ± 0.43	4.80 ± 0.40	0.04
EF% zero time	36.39 ± 7.07	34.13 ± 5.57	0.23
EF% end of follow up	38.16 ± 7.86	31.53 ± 8.74	0.003
ΔEF%	1.74 ± 6.26	2.60 ± 8.82	0.02
N^o^ Hospitalizations	0.86 ± 1.00	1.86 ± 1.50	0.001
No. AHF	0.40 ± 0.71	1.60 ± 1.63	0.000004
NYHA-FC zero	3.16 ± 0.71	3.93 ± 0.25	0.00007
NYHA-FC end	2.33 ± 0.75	3.93 ± 0.25	0.00000
NYHA score	0.25 ± 0.50	1.00 ± 0.37	0.000000
SBP (mmHg)	104.83 ± 15.21	82.30 ± 10.40	0.000000
DBP (mmHg)	68.95 ± 9.04	54.44 ± 6.69	0.000000
Age	57.20 ± 8.72	58.33 ± 7.79	0.637

**Table 3:** Comparison of outcome between the control and the therapy group

a)	Mortality					

*CER*	*EER*	RR	95%CI	RRR%	X^2^	NNT

6/34 0.17	9/79 0.113	0.66	0.21 - 1.96	34%	R=0.37	17.5

b)	Combined outcome					

CER	EER	RR	95%CI	RRR%	X^2^	NNT

29/34 0.85	41/79 0.51	0.6	0.33 - 1.13	40%	R=0.0008	3.03

CER - Control event rate; EER - Experimental event rate; RR - Risk ratio; RRR- Relative risk reduction; NNT - Number needed to treat.

**Table 4:** Comparison of outcome between the control group and the Metoprolol subgroup

a)	Mortality					

CER	EER	RR	95%CI	RRR%	H^2^	NNT

6/34 0.17	4/29 0.13	0.76	0.2 - 3.04	24%	0.68	25

b)	Combined outcome					

CER	EER	RR	95%CI	RRR%	X^2^	NNT

29/34 0.85	20/29 0.68	0.80	0.38 - 1.72	20%	0.0007	5.8

**Table 5:** Comparison of outcome between the control group and the Bisoprolol subgroup

a)	Mortality					

CER	EER	RR	95%CI	RRR%	X^2^	NNT

6/34 0.17	3/24 0.12	0.70	0.16 - 3.12	30%	0.58	20

b)	Combined outcome					

CER	*EER*	RR	95%CI	RRR%	X^2^	NNT

29/34 0.85	14/24 0.58	0.68	0.3 - 1.56	32%	0.022	3.7

**Table 6:** Comparison of outcome between the control group and the Carvedilol subgroup

a)	Mortality					

*CER*	EER	RR	95%CI	RRR%	X^2^	NNT

6/34 0.17	2/26 0.076	0.44	0.08 - 2.34	56%	0.22	11

b)	Combined outcome					

CER	EER	RR	95%CI	RRR%	X^2^	NNT

29/34 0.85	14/26 0.53	0.62	0.28 - 1.43	38%	0.0079	3.1

## Discussion

Our study did not show a statistically significant difference for cardiovascular mortality when comparing conventional therapy with treatment with beta blockers (p = 0.37), but there was a 34% risk reduction for mortality which, on the other hand, is of clinical relevance. Our results for mortality in patients with heart failure when treated with beta blockers are similar to those in the Cibis II study (treatment with Bisoprolol) [6]. Our therapy group showed a significant improvement in the combined outcome (p = 0.0008), RRR 40% and NNT 3.03, which is consistent mostly with the US Carvedilol Study where RRR% for the combined outcome was 38% [7-9].

In the analysis of the mortality and the combined outcome between the control group with each therapy subgroup separately, it appeared that the Metoprolol subgroup did not have a significant difference compared to the control group for mortality (p = 0.68) and RRR was 24%, but for the combined outcome, there was a statistically significant improvement (p = 0.0007), which is consistent with the MCD Study for Metoprolol where the mortality rate was also not significant (p = 0.69) [7].

The Bisoprolol group also did not show a statistically significant difference for mortality compared to the control group (p = 0.58), RRR was 30%, but it showed a significant difference for the combined outcome (p = 0.02) and was RRR 32%. This result differed from the CibisII study where the difference for the mortality was statistically significant, but CibisII study included larger number of patients and lasted longer (two and a half years) [6].

The Carvedilol subgroup compared to the control group did not show a significant difference in mortality (p = 0.220), but RRR was highest among all the subgroups, 56%, and there was also a significant reduction in the combined outcome (p = 0.007), RRR 38%, which was consistent with the US Carvedilol Study where the RRR for the combined outcome was 63% (p = 0.001) [8, 9].

The meta-analysis of Chatterjee (BMJ, 2013) on the effect of beta-blockers showed that they reduced the mortality in heart failure patients, but as a result of their class effect. No specific beta blocker showed predominant effect upon risk reduction of mortality in chronic heart failure patients [9, 10].

In conclusion, our study confirmed that patients with stable chronic heart failure, treated with optimal doses of beta-blockers, had a significant reduction of the risk from death as well as the combined outcome (death and/or number of hospitalizations). No specific beta-blocker surpasses the effect of the other beta-blockers in the treatment of chronic heart failure considering the risk reduction.
